# Foundations of human spatial problem solving

**DOI:** 10.1038/s41598-023-28834-3

**Published:** 2023-01-27

**Authors:** Noah Zarr, Joshua W. Brown

**Affiliations:** Department of Psychological and Brain Sciences, Indiana University, Bloomington, USA

**Keywords:** Cognitive neuroscience, Computational neuroscience

## Abstract

Despite great strides in both machine learning and neuroscience, we do not know how the human brain solves problems in the general sense. We approach this question by drawing on the framework of engineering control theory. We demonstrate a computational neural model with only localist learning laws that is able to find solutions to arbitrary problems. The model and humans perform a multi-step task with arbitrary and changing starting and desired ending states. Using a combination of computational neural modeling, human fMRI, and representational similarity analysis, we show here that the roles of a number of brain regions can be reinterpreted as interacting mechanisms of a control theoretic system. The results suggest a new set of functional perspectives on the orbitofrontal cortex, hippocampus, basal ganglia, anterior temporal lobe, lateral prefrontal cortex, and visual cortex, as well as a new path toward artificial general intelligence.

## Introduction

Great strides have been made recently toward solving hard problems with deep learning, including reinforcement learning^1, 2^. While these are groundbreaking and show superior performance over humans in some domains, humans nevertheless exceed computers in the ability to find creative and efficient solutions to novel problems, especially with changing internal motivation values^3^. Artificial general intelligence (AGI), especially the ability to learn autonomously to solve arbitrary problems, remains elusive^4^.

Value-based decision-making and goal-directed behavior involve a number of interacting brain regions, but how these regions might work together computationally to generate goal directed actions remains unclear. This may be due in part to a lack of mechanistic theoretical frameworks^5, 6^. The orbitofrontal cortex (OFC) may represent both a cognitive map^7^ and a flexible goal value representation^8^, driving actions based on expected outcomes^9^, though how these guide action selection is still unclear. The hippocampus is important for model-based planning^10^ and prospection^11^, and the striatum is important for action selection^12^. Working memory for visual cues and task sets seems to depend on the visual cortex and lateral prefrontal regions, respectively^13, 14^.

Neuroscience continues to reveal aspects of how the brain might learn to solve problems. Studies of cognitive control highlight how the brain, especially the prefrontal cortex, can apply and update rules to guide behavior^15, 16^, inhibit behavior^17^, and monitor performance^18^ to detect and correct errors^19^. Still, there is a crucial difference between rules and goals. Rules define a mapping from a stimulus to a response^20^, but goals define a desired state of the individual and the world^21^. When cognitive control is re-conceptualized as driving the individual to achieve a desired state, or set point, then cognitive control becomes a problem amenable to control theory.

Control theory has been applied to successfully account for the neural control of movement^22^ and has informed various aspects of neuroscience research, including work in *C. Elegans*^23^, and work on controlling states of the brain^24^ and electrical stimulation placement methods^25^ (as distinct from behavioral control over states of the world in the present work), and more loosely in terms of neural representations underlying how animals control an effector via a brain computer interface^26^. In Psychology, Perceptual Control Theory has long maintained that behavior is best understood as a means of controlling perceptual input in the sense of control theory^27, 28^.

In the control theory framework, a preferred decision prospect will define a set point, to be achieved by control-theoretic negative feedback controllers^29, 30^. Problem solving then requires 1) defining the goal state; 2) planning a sequence of state transitions to move the current state toward the goal; and 3) generating actions aimed at implementing the desired sequence of state transitions.

Algorithms already exist that can implement such strategies, including the Dijkstra and A* algorithms^31, 32^ and are commonly used in GPS navigation devices found in cars and cell phones. Many variants of reinforcement learning solve a specific case of this problem, in which the rewarded states are relatively fixed, such as winning a game of Go^33^. While deep Q networks^1^ and generative adversarial networks with monte carlo tree search^33^ are very powerful, what happens when the goals change, or the environmental rules change? In that case, the models may require extensive retraining. The more general problem requires the ability to dynamically recalculate the values associated with each state as circumstances, goals, and set points change, even in novel situations.

Here we explore a computational model that solves this more general problem of how the brain solves problems with changing goals^34^, and we show how a number of brain regions may implement information processing in ways that correspond to specific model components. While this may seem an audacious goal, our previous work has shown how the GOLSA model can solve problems in the general sense of causing the world to assume a desired state via a sequence of actions, as described above^34^. The model begins with a core premise: the brain constitutes a control-theoretic system, generating actions to minimize the discrepancy between actual and desired states. We developed the Goal-Oriented Learning and Selection of Action (GOLSA) computational neural model from this core premise to simulate how the brain might autonomously learn to solve problems, while maintaining fidelity to known biological mechanisms and constraints such as localist learning laws and real-time neural dynamics. The constraints of biological plausibility both narrow the scope of viable models and afford a direct comparison with neural activity.

The model treats the brain as a high-dimensional control system. It drives behavior to maintain multiple and varying control theoretic set points of the agent’s state, including low level homeostatic (e.g. hunger, thirst) and high level cognitive set points (e.g. a Tower of Hanoi configuration). The model autonomously learns the structure of state transitions, then plans actions to arbitrary goals via a novel hill-climbing algorithm inspired by Dijkstra’s algorithm^32^. The model provides a domain-general solution to the problem of solving problems and performs well in arbitrary planning tasks (such as the Tower of Hanoi) and decision-making problems involving multiple constraints^34^ (“Methods”).

**Figure 1:**
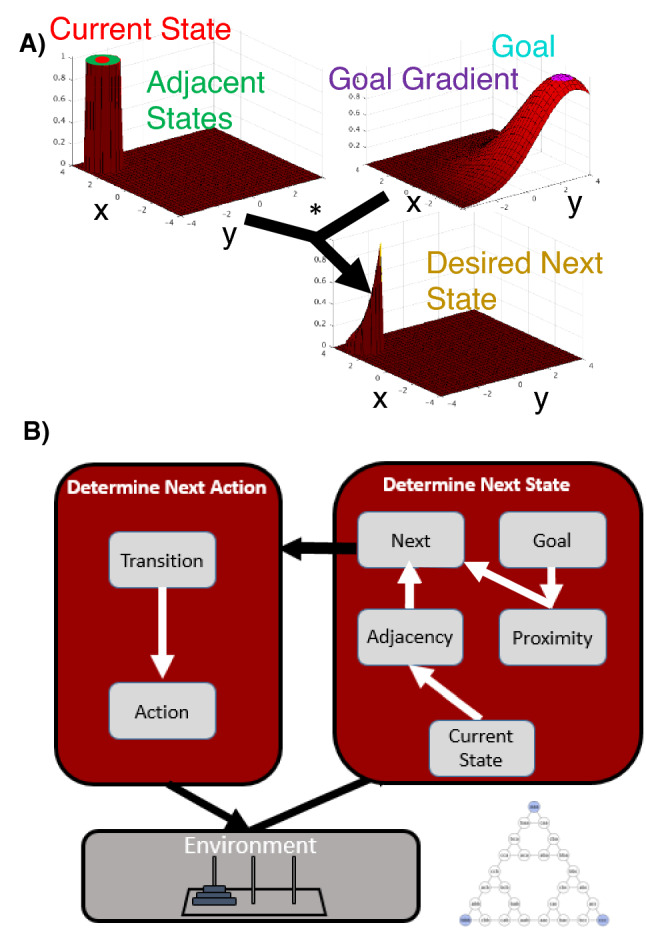
(**A**) The GOLSA model determines the next desired state by hill climbing. Each layer represents the same set of states, one per neuron. The x- and y-axes of the grids represent abstracted coordinates in a space of states. Neurons are connected to each other for states that are reachable from another by one action, in this case neighbors in the x,y plane. The Goal state is activated and spreads activation through a Goal Gradient (Proximity) layer, thus dynamically specifying the value of each state given the goal, so that value is greater for states nearer the goal state. The Current State representation activates all Adjacent States, i.e. that can be achieved with one state transition. These adjacent states mask the Goal Gradient input to the Desired Next State, so that the most active unit in the Desired Next State represents a state attainable with one state transition and which will bring the state most directly toward the goal state. The black arrows indicate that the Desired Next State unit activities are the element-wise products of the corresponding Adjacent States and Goal Gradient unit activities. The font colors match the model layer to corresponding brain regions in Figs. 3 and 4. (**B**) The desired state transition is determined by the conjunction of current state and desired next state. The GOLSA model learns a mapping from desired state transitions to the actions that cause those transitions. After training, the model can generate novel action sequences to achieve arbitrary goal states. Adapted from^34^.

The GOLSA model works by representing each possible state of the agent and environment in a network layer, with multiple layers each representing the same sets of states (Fig. 1A,B). The Goal Gradient layer is activated by an arbitrarily specified desired (Goal) state and spreads activation backward along possible state transitions represented as edges in the network^35, 36^. This value spreading activation generates current state values akin to learned state values (Q values) in reinforcement learning, except that the state values can be reassigned and recalculated dynamically as goals change. This additional flexibility allows goals to be specified dynamically and arbitrarily, with all state values being updated immediately to reflect new goals, thus overcoming a limitation of current RL approaches. Essentially, the Goal Gradient is the hill to climb to minimize the discrepancy between actual and desired states in the control theoretic sense. In parallel, regarding the present state of the model system, the Adjacent States layer receives input from a node representing the current state of the agent and environment, which in turn activates representations of all states that can be achieved with one state transition. The valid adjacent states then mask the Goal Gradient layer to yield the Desired Next State representation. In this layer, the most active unit represents a state which, if achieved, will move the agent one step closer to the goal state. This desired next state is then mapped onto an action (i.e. a controller signal) that is likely to effect the desired state transition. In sum, the model is given an arbitrarily specified goal state and the actual current state of the actor. It then finds an efficient sequence of states to transit in order to reach the goal state, and it generates actions aimed at causing the current state of the world to be updated so that it approaches and reaches the goal state.

Here we test whether and how the GOLSA model might provide an account of how various brain regions work together to drive goal-directed behavior. To do this, we ask human subjects to perform a multi-step task to achieve arbitrary goals. We then train the GOLSA model to perform the same task, and we use representational similarity analysis (RSA) to ask whether specific GOLSA model layers show similar representations to specific brain regions (Supplementary Material). The results will provide a tentative account of the function of specific brain regions in terms of the GOLSA model, and this account can then be tested and compared against alternative models in future work.

### Study design

#### Model

**Figure 2:**
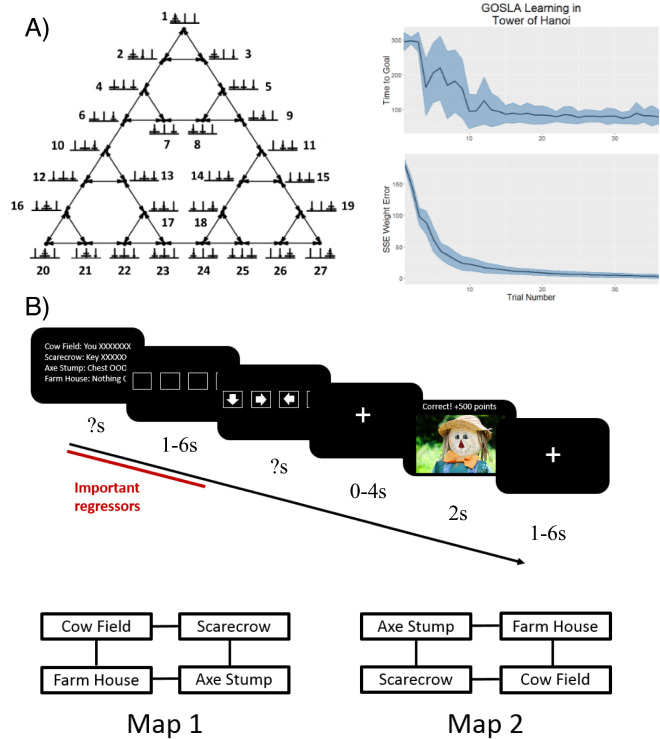
(**A**) The GOLSA model learns to solve problems, achieving arbitrary goal states. It does this by making arbitrary actions and observing which actions cause which state transitions. Figure adapted from earlier work^34, 37^. (**B**) Treasure Hunt task. Both the GOLSA model and the human fMRI subjects performed a simple treasure hunt task, in which subjects were placed in one of four possible starting locations, then asked to generate actions to reach any of the other possible locations. To test multi-step transitions, subjects had to first move to the location of a key needed to unlock a treasure chest, then move to the treasure chest location. Participants first saw an information screen specifying the contents of each of the four states (‘you’, ‘key’, ‘chest’, or ‘nothing’). After a jittered delay, participants selected a desired movement direction and after another delay saw an image of the outcome location. The mapping of finger buttons to game movements was random on each trial and revealed after subjects were given the task and had to plan their movements, thus avoiding motor confounds during planning. Bottom: The two state-space maps used in the experiment. One map was used in the first half of trials while the other was used in the second half, in counterbalanced order.

The details of the model implementation and the model code are available in the “Methods”. Behaviorally, we found that the GOLSA model is able to learn to solve arbitrary problems, such as reaching novel states in the Tower of Hanoi task (Fig. 2A). It does this without hard-wired knowledge, simply by making initially random actions and learning from the outcomes, then synthesizing the learned information to achieve whatever state is specified as the goal state.

Having found that the model can learn autonomously to solve arbitrary problems, we then aimed to identify which brain regions might show representations and activity that matched particular GOLSA model layers. To do this, we tested the GOLSA model with a Treasure Hunt task (Fig. 2B and “Methods”), which was performed by both the GOLSA model and human subjects with fMRI. All human subjects research here was approved by the IRB of Indiana University, and subjects gave full informed consent. The human subjects research was performed in accordance with relevant guidelines/regulations and in accordance with the Declaration of Helsinki. Subjects were placed in one of four starting states and had to traverse one or two states to achieve a goal, by retrieving a key and subsequently using it to unlock a treasure chest for a reward (Fig. 2B). The Treasure Hunt task presents a challenge to standard RL approaches, because the rewarded (i.e. goal) state changes regularly. In an RL framework, the Bellman equation would regularly relearn the value of each possible state in terms of how close it is to the currently rewarded state, forgetting previous state values in the process.

### Representational similarity analysis

To analyze the fMRI and model data, we used model-based fMRI with representational similarity analysis (RSA)^38^ (“Methods”). RSA considers a set of task conditions and asks whether a model, or brain region, can discriminate between the patterns of activity associated with the two conditions, as measured by a correlation coefficient. By considering every possible pairing of conditions, the RSA method constructs a symmetric representational dissimilarity matrix (RDM), where each entry is 1-r, and r is the correlation coefficient. This RDM provides a representational fingerprint of what information is present, so that the fingerprints can be compared between a model layer and a given brain region. For our application of RSA, each representational dissimilarity matrix (RDM) represented the pairwise correlations across 96 total patterns–4 starting states by 8 trial types by 3 time points within a trial (problem description, response, and feedback). For each model layer, the pairwise correlations are calculated with the activity pattern across layer cells in one condition vs. the activity pattern in the same layer in the other condition. For each voxel in the brain, the pairwise correlations are calculated with the activity pattern in a local neighborhood of radius 10 mm (93 voxels total) around the voxel in question, for one condition vs. the other condition. The 10 mm radius was chosen to provide a tradeoff between a sufficiently high number of voxels for pattern analysis and a sufficiently small area to identify specific regions. The fMRI RSA maps are computed for each subject over all functional scans and then tested across subjects for statistical significance. The comparison between GOLSA model and fMRI RDMs consists of looking for positive correlations between elements of the upper symmetric part of a given GOLSA model layer RDM vs. the RDM around a given voxel in the fMRI RDMs. The resulting fMRI RSA maps, one per GOLSA model layer, show which brain regions have representational similarities between particular model components and particular brain regions. The fMRI RSA maps showing the similarities between a given GOLSA model layer and a given brain region are computed for each subject and then tested across subjects for statistical significance in a given brain region, with whole-brain tests for significance in all cases. Full results are in Table 2, and method details are in the “Methods” section. As a control, we also generated a null model layer that consisted of normally distributed noise (μ = 1, σ = 1). In the null model, no voxels exceeded the cluster defining threshold, and so no significant clusters were found, which suggests that the results below are not likely to reflect artifacts of the analysis methods.

## Results

### Orbitofrontal cortex, goals, and maps

**Figure 3:**
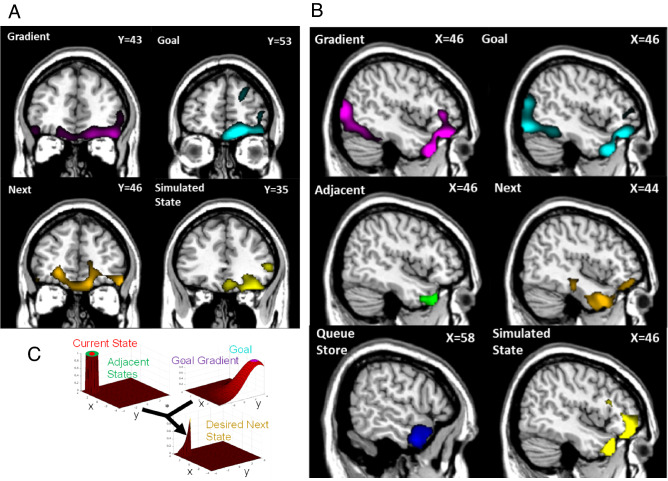
Representational Similarity Analysis (RSA) of model layers vs. human subjects performing the same Treasure Hunt task. All results shown are significant clusters across the population with a cluster defining threshold of *p* < 0.001 cluster corrected to *p* < 0.05 overall, and with additional smoothing of 8 mm FWHM applied prior to the population level t-test for visualization purposes. (**A**) population Z maps showing significant regions of similarity to model layers in orbitofrontal cortex. Cf. Figure 1 and Fig. 5B. The peak regions of similarity for goal-gradient and goal show considerable overlap in right OFC. The region of peak similarity for simulated-state is more posterior. To most clearly show peaks of model-image correspondence, the maps of gradient and goal are here visualized at *p* < 0.00001 while all others are visualized at *p* < 0.001. (**B**) Z maps showing significant regions of similarity to model layers in right temporal cortex. The peak regions of similarity for goal-gradient and goal overlap and extend into the OFC. The peak regions of similarity for adjacent-states, next-desired-state, and -simulated-state occur in similar but not completely overlapping regions, while the cluster for queue-store is more lateral. (**C**) Fig. 1A, copied here as a legend, where the font color of each layer name corresponds to the region colors in panels (**A)** and (**B)**.

**Figure 4:**
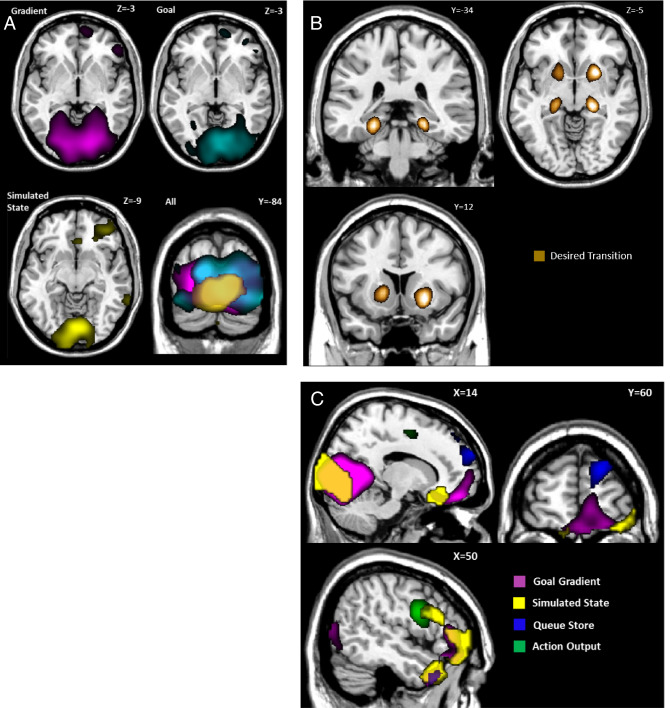
Representational Similarity Analysis of model layers vs. human subjects performing the same Treasure Hunt task, with the same conditions and RSA analysis as in Fig. 3. (**A**) Population Z maps showing significant regions of similarity to model layers in visual cortex. The peak regions of similarity for goal-gradient and goal overlap substantially, primarily in bilateral cuneus, inferior occipital gyrus, and lingual gyrus. The simulated-state layer displayed significantly similar activity to that in a smaller medial and posterior region. Statistical thresholding and significance are the same as Fig. 3. (**B**) Z map showing significant regions of similarity to the desired-transition layer. Similarity peaks were observed for desired-transition in bilateral hippocampal gyrus as well as bilateral caudate and putamen. The desired-transition map displayed here was visualized at *p* < 0.00001 for clarity. (**C**) Z maps showing significant regions of similarity to the model layers in frontal cortex. Similarity peaks were observed for queue-store in superior frontal gyrus (BA10). Action-output activity most closely resembled activity in inferior frontal gyrus (BA9), while simulated-state and goal-gradient patterns of activity were more anterior (primarily BA45). Similarity between activity in the latter two layers and activity in OFC, visual cortex, and temporal pole is also visible.

We found that the patterns of activity in a number of distinct brain regions match those expected of a control theoretic system, as instantiated in the GOLSA model (Figs. 3A,B and 4A–C); Table 1). Orbitofrontal cortex (OFC) activity patterns match model components that represent both a cognitive map^7^ and a flexible goal value representation^8^, specifically matching the Goal and Goal Gradient layer activities. These layers represent the current values of the goal state and the current values of states near the goal state, respectively. The Goal Gradient layer incorporates cognitive map information in terms of which states can be reached from which other states. This suggests mechanisms by which OFC regions may calculate the values of states dynamically as part of a value-based decision process, by spreading activation of value from a currently active goal state representation backward. The GOLSA model representations of the desired next state also match overlapping regions in the orbitofrontal cortex (OFC) and ventromedial prefrontal cortex (vmPFC), consistent with a role in finding the more valuable decision option (Fig. 3). Reversal learning and satiety effects as supported by the OFC reduce to selecting a new goal state or deactivating a goal state respectively, which immediately updates the values of all states. Collectively this provides a mechanistic account of how value-based decision-making functions in OFC and vmPFC.

**Table 1:** Significant similarity clusters for RSA analysis. The p and Size columns refer to cluster-corrected values. Anatomical labels are derived from the Automated Anatomical Labeling Atlas in SPM5^46^.

Layer	MNI coordinates							
	Peak region (TD label)	X	Y	Z	Z-Score	Peak R	*p*	Size
goal	Cuneus/frontal	− 14	− 86	14	6.75	0.0284	< 0.001	12,209
goal-gradient	Cuneus/frontal	17	− 79	− 7	7.70	0.0414	< 0.001	11,528
goal-gradient	Postcentral gyrus	− 21	− 38	68	4.34	0.0127	0.001	136
goal-gradient	Superior frontal gyrus	− 28	45	34	3.77	0.0115	0.012	86
goal-gradient	Middle temporal gyrus	72	− 31	− 3	4.31	0.0105	0.007	97
adjacent-state	Middle temporal pole	45	24	− 37	3.54	0.0205	0.030	79
next-desired-state	Medial frontal gyrus	14	31	− 14	5.09	0.0143	< 0.001	1072
next-desired-state	Putamen	28	− 10	14	4.24	0.0125	0.035	67
next-desired-state	Superior temporal gyrus	− 34	10	− 34	4.69	0.0077	< 0.001	226
next-desired-state	Superior temporal gyrus	31	14	− 41	4.49	0.0128	< .001	616
next-desired-state	Pons	3	− 28	− 37	4.15	0.0110	< .001	227
next-desired-state	Precuneus	− 17	− 48	37	3.87	0.0045	0.019	79
desired-transition	Striatum	− 24	− 7	37	6.82	0.0846	< 0.001	5615
desired-transition	Posterior cingulate	− 24	− 72	10	4.63	0.0575	0.012	132
desired-transition	Cerebellum	− 7	− 58	− 34	4.36	0.0235	< .001	256
desired-transition	Occipital lobe	28	− 58	− 3	4.30	0.0298	< .001	267
desired-transition	Precuneus	− 17	− 58	41	4.25	0.0543	0.036	96
action-output	Precentral gyrus	− 41	0	34	5.29	0.0240	0.001	354
action-output	Middle frontal gyrus	28	− 7	44	5.06	0.0216	< 0.001	908
action-output	Supramarginal gyrus	− 38	− 45	37	4.48	0.0174	0.002	147
queue-store	Superior frontal gyrus	21	58	27	4.15	0.0210	0.002	173
queue-store	Middle temporal gyrus	52	3	− 34	4.43	0.0178	< .001	340
queue-store	Temporal lobe	34	− 45	− 10	4.36	0.0068	0.048	81
queue-store	Postcentral gyrus	− 58	− 17	17	3.95	0.0274	0.009	130
simulated-state	Lingual gyrus	3	− 83	− 7	5.15	0.0885	< 0.001	1381
simulated-state	Superior temporal pole	45	24	− 31	4.60	0.0525	< 0.001	795
simulated-state	Middle frontal gyrus	− 52	45	− 3	3.81	0.0341	0.001	388
simulated-state	Middle frontal gyrus	69	− 41	− 3	4.11	0.0410	0.014	92
state	Extra-nuclear	− 31	− 14	24	7.78	0.0191	< 0.001	43,492

### Lateral PFC and planning

**Figure 5:**
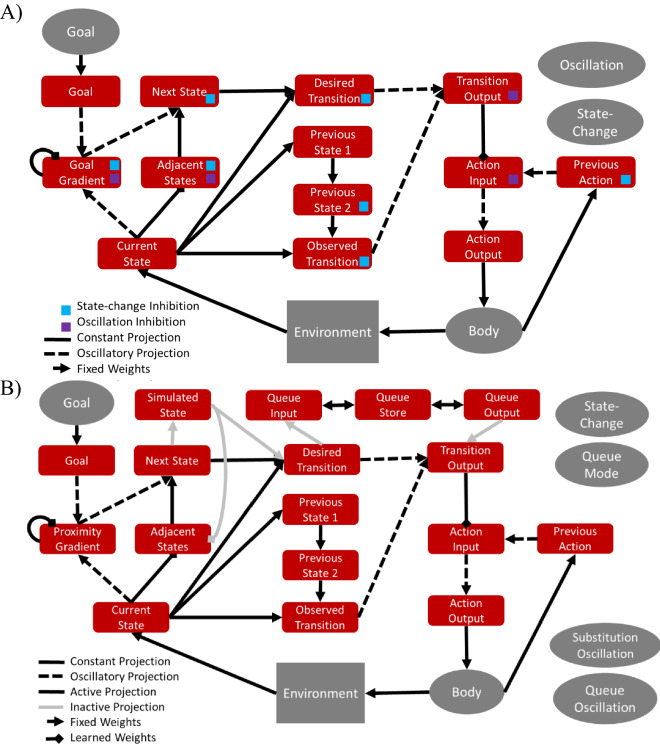
(**A**) Full diagram of core model. Each rectangle represents a layer and each arrow a projection. The body is a node, and two additional nodes are not shown which provide inhibition at each state-change and oscillatory control. The colored squares indicate which layers receive inhibition from these nodes. Some recurrent connections not shown. (**B**) Full diagram of extended model, with added top row representing ability to plan multiple state transition steps ahead (Simulated State, Queue Input, Queue Store, and Queue Output layers). Adapted with permission from earlier work^34^.

The GOLSA model also incorporates a mechanism that allows multi-step planning, by representing a Simulated State as if the desired next state were already achieved, so that the model can plan multiple subsequent state transitions iteratively prior to committing to a particular course of action (Fig. 5B). Those subsequent state transitions are represented in a Queue Store layer pending execution via competitive queueing, in which the most active action representation is the first to be executed, followed by the next most active representation, and so on^39, 40^. This constitutes a mechanism of prospection^41^ and planning^42^. The Simulated State layer in the GOLSA model shows strong representational similarity with regions of the OFC and anterior temporal lobe, and the Queue Store layer shows strong similarity with the anterior temporal lobe and lateral prefrontal cortex. This constitutes a mechanistic account of how the vmPFC and OFC in particular might contribute to multi-step goal-directed planning, and how plans may be stored in lateral prefrontal cortex.

### Visual cortex and future visual states

The visual cortex also shows representational patterns consistent with representing the goal, goal gradient, and simulated future states (Figs. 3B and 4). This suggests a role for the visual cortex in planning, in the sense of representing anticipated future states beyond simply representing current visual input. Future states in the present task are represented largely by images of locations, such as an image of a scarecrow or a house. In that sense, an anticipated future state could be decoded as matching the representation of the image of that future state. One possibility is that this reflects an attentional effect that facilitates processing of visual cues representing anticipated future states. Another possibility is that visual cortex activity signals a kind of working memory for anticipated future visual states, similar to how working memory for past visual states has been decoded from visual cortex activity^14^. This would be distinct from predictive coding, in that the activity predicts future states, not current states^43^. In either case, the results are consistent with the notion that the visual cortex may not be only a sensory region but may play some role in planning by representing the details of anticipated future states.

### Anterior temporal lobe and planning

The anterior temporal lobe likewise shows representations of the goal, goal gradient, the adjacent states, the next desired state, and simulated future and queue store states (Figs. 3B, 4C). In one sense this is not surprising, as the states of the task are represented by images of objects, and visual objects (especially faces) are represented in the anterior temporal lobe^44^. Still, the fact that the anterior temporal lobe shows representations consistent with planning mechanisms suggests a more active role in planning beyond feedforward sensory processing as commonly understood^45^.

### Hippocampal region and prospection

Once the desired next state is specified, it must be translated to an action. The hippocampus and striatum match the representations of the Desired Transition layer in the GOLSA model. This model layer represents a conjunction of the current state and desired next state transitions, which in the GOLSA model is a necessary step toward selecting an appropriate action to achieve the desired transition. This is consistent with the role of the hippocampus in prospection^41^, and it suggests computational and neural mechanisms by which the hippocampus may play a key role in turning goals into predictions about the future, for the purpose of planning actions^10, 11^. Finally, as would be expected, the motor output representations in the GOLSA model match motor output patterns in the motor cortex (Fig. 4C).

## Discussion

The results above show how a computational neural model, the GOLSA model, provides a novel computational account of a number of brain regions. The guiding theory is that a substantial set of brain regions function together as a control-theoretic mechanism^47^, generating behaviors to minimize the discrepancy between the current state and the desired (goal) state. The OFC is understood as including neurons that represent the value of various states in the world, such as the value of acquiring certain objects. Greater activity of an OFC neuron corresponds with more value of its represented state given the current goals. Because of spreading activation, neurons will be more active if they represent states closer to the goal. This results in value representations similar to those provided by the Bellman equation of reinforcement learning^48^, with the difference being that spreading activation can instantly reconfigure the values of states as goals change, without requiring extensive iterations of the Bellman equation.

Given the current state and the goal state, the next desired state can be determined as a nearby state that can be reached and that also moves the current state of the world closer to the goal state. Table 1 shows these effects in the medial frontal gyrus, putamen, superior temporal gyrus, pons, and precuneus. The GOLSA model suggests this is computed as the activation of available state representations, multiplied by the OFC value for that state. Precedent for this kind of multiplicative effect has been shown in the attention literature^49^. The action to be generated is represented by neural activity in the motor cortex region. This in turn is determined on the basis of neurons that are active specifically for a conjunction of the particular current state and next desired state. Neurally, we find this conjunction represented across a large region including the striatum and hippocampus. This is consistent with the notion of the hippocampus as a generative recurrent neural network, that starts at a current state and runs forward, specifically toward the desired state^50^. The striatum is understood as part of an action gate that permits certain actions in specific contexts, although the GOLSA model does not include an explicit action gate^51^. Where multiple action steps must be planned prior to executing any of them, the lateral PFC seems to represent a queue of action plans in sequence, as sustained activity representing working memory^39, 52^. By contrast, working memory representations in the visual cortex apparently represent the instructed future states as per the instructions for each task trial, and these are properly understood as visual sensory rather than motor working memories^14^.

Our findings overall bear a resemblance to the Free Energy principle. According to this, organisms learn to generate predictions of the most likely (i.e. rewarding) future states under a policy, then via active inference emit actions to cause the most probable outcomes to become reality, thus minimizing surprise^53, 54^. Like active inference, the GOLSA model emits actions to minimize the discrepancy between the actual and predicted state. Of note, the GOLSA model specifies the future state as a desired state rather than a most likely state. This crucial distinction allows a state that has a high current value to be pursued, even if the probability of being in that state is very low (for example buying lottery tickets and winning). Furthermore, the model includes the mechanisms of Fig. 1, which allow for flexible planning given arbitrary goals. The GOLSA model is a process model and simulates rate-coded neural activity as a dynamical system (“Methods”), which affords the more direct comparison with neural activity representations over time as in Figs. 3 and 4.

The GOLSA model, and especially our analysis of it, builds on recent work that developed methods to test computational neural models against empirical data. Substantial previous work has demonstrated how computational neural modeling can provide insight into the functional properties underlying empirical neural data, such as recurrent neural networks elucidating the representational structure in anterior cingulate^19, 55, 56^ and PFC^57^; deep neural networks accounting for object recognition in IT with representational similarity analysis^58^, and encoding/decoding of visual cortex representations^59^; dimensionality reduction for comparing neural recordings and computational neural models^60^, and representations of multiple learned tasks in computational neural models^61^.

The GOLSA model shares some similarity with model-based reinforcement learning (MBRL), in that both include learned models of next-state probabilities as a function of current state and action pairs. Still, a significant limitation of both model-based and model free RL is that typically there is only a single ultimate goal, e.g. gaining a reward or winning a game. Q-values^62^ are thus learned in order to maximize a single reward value. This implies several limitations: (1) that Q values are strongly paired with corresponding states; (2) that there is only one Q value per state at a given time, as in a Markov decision process (MDP), and (3) Q values are generally updated via substantial relearning. In contrast, real organisms will find differing reward values associated with different goals at different times and circumstances. This implies that goals will change over time, and re-learning Q-values with each goal change would be inefficient. Instead, a more flexible mechanism will dynamically assign values to various goals and then plan accordingly. The GOLSA model exemplifies this approach, essentially replacing the learned Q values of MBRL and MDPs with an activation-based representation of state value, which can be dynamically reconfigured as goals change. This overcomes the three limitations above.

Our work has several limitations. First, regarding the GOLSA model itself, the main limitation is its present implementation of one-hot state representations. This makes a scale-up to larger and continuous state spaces challenging. Future work may overcome this limitation by replacing the one-hot representations with vector-valued state representations and the learned connections with deep network function approximators. This would require corresponding changes in the search mechanisms of Fig. 1A, from parallel, spreading activation to a serial, monte carlo tree search mechanism. This would be consistent with evidence of serial search during planning^63, 64^ and would afford a new approach to artificial general intelligence that is both powerful and similar to human brain function. Another limitation is that the Treasure Hunt task is essentially a spatial problem solving task. We anticipate that the GOLSA model could be applied to solve more general, non-spatial problems, but this remains to be demonstrated.

The fMRI analysis here has several limitations as well. First, a correspondence of representations does not imply a correspondence of computations, nor does it prove the model correct in an absolute sense^65^. There are other computational models that use diffusion gradients to solve goal-directed planning^66^, and more recent work with deep networks to navigate from arbitrary starting to arbitrary ending states^50^. The combined model and fMRI results here constitute a proposed functional account of the various brain regions, but our results do not prove that the regions compute exactly what the corresponding model regions do, nor can we definitively rule out competing models. Nevertheless the ability of the model to account for fMRI data selectively in specific brain regions suggests that it merits further investigation and direct tests against competing models, as a direction for future research. Future work might compare other models besides GOLSA against the fMRI data using RSA, to ascertain whether other model components might provide a better fit to, and account of, specific brain regions. While variations of model-based and model-free reinforcement learning models would seem likely candidates, we know of only one model by Banino et al.^50^ endowed with the ability to flexibily switch goals and thus perform the treasure hunt task as does the GOLSA model^34^. It would be instructive to compare the overall abilities of GOLSA and the model of Banino et al. to account the RDMs of specific brain regions in the Treasure Hunt task, although it is unclear how to do a direct comparison given that the two models consist of very different mechanisms.

The GOLSA model may in principle be extended hierarchically. The frontal cortex has a hierarchical representational structure, in which higher levels of a task may be represented as more anterior^67^. Such hierarchical structure has been construed to represent higher, more abstract task rules^13, 15, 68^. The GOLSA model suggests another perspective, that higher level representations consist of higher level goals instead of higher level rules. In the coffee-making task for example^69^, the higher level task of making coffee may require a lower level task of boiling water. If the GOLSA model framework were extended hierarchically, the high level goal of having coffee prepared would activate a lower level goal of having the water heated to a specified temperature. The goal specification framework here is intrinsically more robust than a rule or schema based framework–rules may fail to produce a desired outcome, but if an error occurs in the GOLSA task performance, replanning simply calculates the optimal sequence of events from whatever the current state is, and the error will be automatically addressed.

This incidentally points to a key difference between rules and goals, in that task rules define a mapping from stimuli to responses^15^, in a way that is not necessarily teleological. Goals, in contrast, are by definition teleological. This distinction roughly parallels that between model-free and model-based reinforcement learning^70^ The rule concept, as a stimulus–response mapping, implies that an error is a failure to generate the action specified by the stimulus, regardless of the final state of a system. In contrast, the goal concept implies that an error is precisely a failure to generate the desired final state of a system. Well-learned actions may acquire a degree of automaticity over time^71^, but arguably the degree of automaticity is independent of whether an action is rule oriented vs. goal-directed. If a goal-directed action becomes automatized, this does not negate the teleological nature, namely that errors in the desired final state of the world can be detected and lead to corrective action to achieve the desired final state. Rule-based action, whether deliberate or automatized, does not necessarily entail corrective action to achieve a desired state. Where actions are generated, and possibly corrected, to achieve a desired state of the world, this may properly be referred to as goal-directed behavior.

## Conclusion

We have investigated the GOLSA model here to examine whether and how it might account for the function of specific brain regions. With RSA analysis, we found that specific layers of the GOLSA model show strong representational similarities with corresponding brain regions. Goals and goal value gradients matched especially the orbitofrontal cortex, and also some aspects of the visual and anterior temporal cortices. The desired transition layer matched representations in the hippocampus and striatum, and simulated future states matched representations in the middle frontal gyrus and superior temporal pole. Not surprisingly, the model motor layer representations matched the motor cortex. Collectively, these results constitute a proposal that the GOLSA model can provide an organizing account of how multiple brain regions interact to form essentially a negative feedback controller, with time varying behavioral set points derived from motivational states. Future work may investigate this proposal in more depth and compare against alternative models.

## Methods

### Model components

#### Layers

The GOLSA model is constructed from a small set of basic components, and the model code is freely available as supplementary material. The main component class is a layer of units, where each unit represents a neuron (or, more abstractly, a small subpopulation of neurons) corresponding to either a state, a state transition, or an action. The activity of units in a layer represents the neural firing rate and is instantiated as a vector updated according to a first order differential equation (c.f. Grossberg^72^). The activation function varies between layers, but all units in a particular layer are governed by the same equation. The most typical activation function for a single unit is,

1 $$da\left(t\right)=\frac{1}{\tau }\left(-\lambda a\left(t\right)dt+\left(1-a\left(t\right)\right)Edt-Idt+ \varepsilon N\left(t\right)\sqrt{dt}\right),$$

where *a* represents activation, i.e. the firing rate, of a model neuron. The four terms of this equation represent, in order: passive decay $-\lambda a(t)$, shunting excitation $\left(1-a\left(t\right)\right)E$, linear inhibition $-I$, and random noise $\varepsilon N(t)\sqrt{dt}$. “Shunting” refers to the fact that excitation (E) scales inversely as current activity increases, with a natural upper bound of 1. The passive decay works in a similar fashion, providing a natural lower bound activity of 0. The inhibition term linearly suppresses unit activity, while the final term adds normally distributed noise N (μ = 0, σ = 1), with strength $\varepsilon$. Because the differential equations are approximated using the Euler method, the noise term is multiplied by $\sqrt{dt}$ to standardize the magnitude across different choices of dt^73, 74^. The speed of activity change is determined by a time constant τ. The parameters τ, λ, ε vary by layer in order to implement different processes. E and I are the total excitation and inhibition, respectively, impinging on a particular unit for every presynaptic unit j in every projection p onto the target unit,

2 $$E= \sum_{p}\sum_{j}{[w}_{{p}_{j}}{a}_{{p}_{j}}{]}^{+}$$

3 $$I= \sum_{p}\sum_{j}{[w}_{{p}_{j}}{a}_{{p}_{j}}{]}^{-}$$

where ${a}_{{p}_{j}}$ is the activation of a presynaptic model neuron that provides exciation, and ${w}_{{p}_{j}}$ is the synaptic weight that determines how much excitation per unit of presynaptic activity will be provided to the postsynaptic model neuron.

A second activation function used in several places throughout the model is,

4 $$da(t)=\frac{1}{\tau }\left(-\lambda a(t)dt+\left(1-a\left(t\right)\right)Edt-a(t)Idt+ \varepsilon N(t)\sqrt{dt}\right)$$

This function is identical to Eq. (1) except that the inhibition is also shunting, such that it exhibits a strong effect on highly active units and a smaller effect as unit activity approaches 0. While more typical in other models, shunting inhibition has a number of drawbacks in the current model. Two common uses for inhibition in the GOLSA model are winner-take-all dynamics and regulatory inhibition which resets layer activity. Shunting inhibition impedes both of these processes because inhibition fails to fully suppress the appropriate units, since it becomes less effective as unit activity decreases.

### Projections

Layers connect to each other via projections, representing the synapses connecting one neural population to another. The primary component of projections is a weight matrix specifying the strength of connections between each pair of units. Learning is instantiated by updating the weights according to a learning function. These functions vary between the projections responsible for the model learning and are fully described in the section below dealing with each learning type. Some projections also maintain a matrix of traces updated by a projection-specific function of presynaptic or postsynaptic activity. The traces serve as a kind of short-term memory for which pre or postsynaptic units were recently activated, which serve a very similar role to eligibility traces as in Barto et al.^75^, though with a different mathematical form.

### Nodes

Nodes are model components that are not represented neurally via an activation function. They represent important control and timing signals to the model and are either set externally or update autonomously according to a function of time. For instance, sinusoidal oscillations are used to gate activity between various layers. While in principle rate-coded model neurons could implement a sinusoidal wave, the function is simply hard coded into the update function of the node for simplicity. In some cases, it is necessary for an entire layer to be strongly inhibited when particular conditions hold true, such as when an oscillatory node is in a particular phase. Layers therefore also have a list of inhibitor nodes that prevent unit activity within the layer when the node value meets certain conditions. In a similar fashion, some projections are gated by nodes such that they allow activity to pass through and/or allow the weights to be updated only when the relevant node activity satisfies a particular condition. Another important node provides strong inhibition to many layers when the agent changes states.

### Environment

The agent operates in an environment consisting of discrete states, with a set of allowable state transitions. Allowable state transitions are not necessarily bidirectional, but for the present simulations, they are deterministic (unlike the typical MDP formulation used in RL). In some simulations, the environment also contains different types of reward located in various states, which can be used to drive goal selection. In other simulations, the goal is specified externally via a node value.

### Complete network

Each component and subnetwork of the model is described in detail below or in the main text, but for reference and completeness a full diagram of the core network is shown in Fig. 5A, and the network augmented for multi-step planning is shown in Fig. 5B. Some of the basic layer properties are summarized in Table 2. Layers and nodes are referred to using italics, such that the layer representing the current state is referred to simply as current-state.

**Table 2:** Full list of core model layers and associated parameters.

Layer name	Inhibition type	Time constant	Decay rate	Noise gain
Current-state	Shunting	0.5	1	0
Goal	Linear	1	1	0
Goal-gradient	Linear	1	1	0
Adjacent-states	Linear	0.2	1	0.01
Next-desired-state	Linear	1	0.1	0
Previous-state-1	Shunting	4	0.5	0
Previous-state-2	Linear	1	0.001	0
Previous-action	Linear	1	0.001	0
Observed-transition	Linear	1.5	1	0
Desired-transition	Linear	1.5	1	0
Transition-output	Linear	1.5	1	0
Action-input	Linear	1	1	0
Action-output	Linear	0.5	0.2	0

### Representational structure

In Fig. 5B, the layers Goal, Goal Gradient, Next State, Adjacent States, Previous States, Simulated State, and Current State all have the same number of nodes and the same representational structure, i.e. one state per node.

The layers Desired Transition, Observed Transition, Transition Output, Queue Input, Queue Output, and Queue Store likewise have the same representational structure, which is the number of possible states squared. This allows a node in these layers to represent a transition from one specific state to another specific state.

The layers Action Input, Action Output, and Previous Action all have the same representational structure, which is one possible action per node.

### Task description

The Treasure Hunt task (Fig. 2) was created and presented in OpenSesame, a Python-based toolbox for psychological task design^76^. In the task, participants control an agent which can move within a small environment comprised of four distinct states. The nominal setting is a farm, and the states are a field with a scarecrow, the lawn in front of the farm house, a stump with an axe, and a pasture with cows. Each is associated with a picture of the scene obtained from the internet. These states were chosen to exemplify categories previously shown to elicit a univariate response in different brain regions, namely faces, houses, tools, and animals^77–79^.

Over the course of the experiment, participants were told the locations of treasure chests and the keys needed to open them. By arriving at a chest with the key, participants earned points which were converted to a monetary bonus at the end of the experiment. The states were arranged in a square, where each state was accessible from the two adjacent states but not the state in the opposite corner (diagonal movement was not allowed).

Each trial began with the presentation of a text screen displaying the relevant information for the next trial, namely the locations of the participant, the key, and the chest (Fig. 2). Because the neural patterns elicited during the presentation were the primary target of the decoding analysis, it was important that visual information be as similar as possible across different goal configurations, to avoid potential confounds. To hold luminance as constant as possible across conditions, each line always had the same number of characters. Since, for instance, “Farm House: key” has fewer characters than “Farm House: Nothing”, filler characters were added to the shorter lines, namely Xs and Os. On some trials Xs were the filler characters on the top row and Os were the filler characters on the bottom rows. This manipulation allowed us to attempt to decode the relative position of the Xs and Os to test whether decoding could be achieved due only to character-level differences in the display. We found no evidence that our results reflect low level visual confounds such as the properties of the filler characters.

Participants were under no time constraint on the information screen and pressed a button when they were ready to continue. A delay screen then appeared consisting of four empty boxes. After a jittered interval (1-6 s, distributed exponentially), arrows appeared in the boxes. The arrows represented movement directions and the boxes corresponded to four buttons under the participants left middle finger, left index finger, right index finger, and right middle finger, from left to right. Participants pressed the button corresponding to the box with the arrow pointing in the desired direction to initiate a movement. A fixation cross then appeared for another jittered delay of 0–4 s, followed by a 2 s display of the newly reached location if their choice was correct or an error screen if it was incorrect.

If the participant did not yet have the key required to open the chest, the correct movement was always to the key. Sometimes the key and chest were in the same location in which case the participant would earn points immediately. If they were in different locations, then on the next trial the participant had to move to the chest. This structure facilitated a mix of goal distances (one and two states away) while controlling the route required to navigate to the goal.

If the chosen direction was incorrect, participants saw an error screen displaying text and a map of the environment. Participants advanced from this screen with a button press and then restarted the failed trial. If the failed trial was the second step in a two-step sequence (i.e., if they had already gotten the key and then moved to the wrong state to get to the chest), they had to repeat the previous two trials.

Repeating the failed trial ensured that there were balanced numbers of each class of event for decoding, since an incorrect response indicated that some information was not properly maintained or utilized. For example, if a participant failed the second step of a two-trial sequence, then they may not have properly encoded the final goal when first presented with the information screen on the previous trial, which specified the location of the key and the chest.

Halfway through the experiment, the map was reconfigured such that states were swapped across the diagonal axes of the map. This was necessary because otherwise, each state could be reached by exactly two movement directions and exactly two movement directions could be made from it. For instance, if the farm house was the state in the lower left, the farmhouse could only be reached by moving left or down from adjacent states, and participants starting at the farm house could only move up or to the right. If this were true across the entire experiment, above-chance classification of target state, for instance, could appear in regions that in fact only contain information about the intended movement direction.

Each state was the starting state for one quarter of the trials and the target destination for a different quarter of the trials. All trials were one of three types. One category consisted of single-trial (single) sequences in which the chest and key were in the same location. The sequences in which the chest and key were in separate locations required two trials to complete, one to move from the initial starting location to the key and another to move from the key location to the chest location. These two steps formed the other two classes of trials, the first-of-two (first) and second-of-two (second) trials. Recall that on second trials, no information other than the participant’s current location is presented on the starting screen to ensure that the participant maintained the location of the chest in memory across the entire two-trial sequence (if it was presented on the second trial, there would be no need to maintain that information through the first trial). The trials were evenly divided into single, first, and second classes with 64 trials in each class. Therefore, every trial had a starting state and an immediate goal, while one third of trials also had a more distant final goal.

Immediately prior to participating in the fMRI version of the task, participants completed a short 16-trial practice outside the scanner to refresh their memory. Before beginning the first run inside the scanner, participants saw a map of the farm states and indicated when they had memorized it before moving on. Within each run, participants completed as many trials as they could within eight minutes. As described above, exactly halfway through the trials, the state space was rearranged with each state moving to the opposite corner. Therefore, when participants completed the first half of the experiment, the current run was terminated and participants were given time to learn the new state space before scanning resumed. At the end of the experiment, participants filled out a short survey about their strategy.

### Participants

In total, 49 participants (28 female) completed the behavioral-only portion of the experiment, including during task piloting (early versions of the behavioral task were slightly different than described below). Participants provided written informed consent in accordance with the Institutional Review Board at Indiana University, and were compensated $10/hour for their time plus a performance bonus based on accuracy up to an additional $10. The behavioral task first served as a pilot during task design and then as a pre-screen for the fMRI portion, in that only participants with at least 90% accuracy were invited to participate. Additional criteria for scanning were that the subjects be right handed, free of metal implants, free of claustrophobia, weigh less than 440 pounds, and not be currently taking psychoactive medication. In total, 25 participants participated in the fMRI task but one subject withdrew shortly after beginning, leaving 24 subjects who completed the imaging task (14 female). Across the 24 subjects, the average error rate of responses during the fMRI task was 2.4%, and error trials were modeled separately in the fMRI analysis. These were not analyzed further as there were too few error trials for a meaningful analysis.

### fMRI acquisition and data preprocessing

Imaging data were collected on a Siemens Magnetom Trio 3.0-Tesla MRI scanner and a 32 channel head coil. Foam padding was inserted around the sides of the head to increase participant comfort and reduce head motion. Functional T2* weighted images were acquired using a multiband EPI sequence^80^ with 42 contiguous slices and 3.44 × 3.44 × 3.4 mm^3^ voxels (echo time = 28 ms; flip angle = 60; field of view = 220, multiband acceleration factor = 3). For the first subject, the TR was 813 ms, but during data collection for the second subject the TR changed to 816 ms for unknown reasons. The scanner was upgraded after collecting data from an additional five subjects, at which point the TR remained constant at 832 ms. All other parameters remained unchanged. High-resolution T_1_–weighted MPRAGE images were collected for spatial normalization (256 × 256 × 160 matrix of 1 × 1 × 1mm^3^ voxels, TR = 1800, echo time = 2.56 ms; flip angle = 9).

Functional data were spike-corrected using AFNI’s 3dDespike (http://afni.nimh.nih.gov/afni). Functional images were corrected for difference in slice timing using sinc-interpolation and head movement using a least-squares approach with a 6-parameter rigid body spatial transformation. For subjects who moved more than 3 mm total or 0.5 mm between TRs, 24 motion regressors were added to subsequent GLM analyses^81^.

Because MVPA and representation similarity analysis (RSA) rely on precise voxelwise patterns, these analyses were performed before spatial normalization. For the univariate analyses, structural data were coregistered to the functional data and segmented into gray and white matter probability maps^82^. These segmented images were used to calculate spatial normalization parameters to the MNI template, which were subsequently applied to the functional data. As part of spatial normalization, the data were resampled to 2 × 2 × 2mm^3^, and this upsampling allowed maximum preservation of information. All analyses included a temporal high-pass filter (128 s) and correction for temporal autocorrelation using an autoregressive AR(1) model.

### Univariate GLM

For initial univariate analyses, we measured the neural response associated with each outcome state at the outcome screen (when an image of the state was displayed), as well as the signal at the start of the trial associated with each immediate goal location. Five timepoints were modeled in the GLM used in this analysis, namely the start of the trial, the button press to advance, the appearance of the arrows and subsequent response, the start of the feedback, and the end of the feedback. The regressors marking the start of the trial and the start of the feedback screen were further individuated by the immediate goal on the trial. A separate error regressor was used when the response was incorrect, meaning they did not properly pursue the immediate goal and received error feedback. All correct trials in which participants moved to, for instance, the cow field, used the same trial start and feedback start regressors.

The GLM was fit to the normalized functional images. The resulting beta maps were combined at the second level with a voxel-wise threshold of *p* < 0.001 and cluster corrected (*p* < 0.05) to control for multiple comparisons. We assessed the univariate response associated with each outcome location, by contrasting each particular outcome location with all other outcome locations. The response to the error feedback screen was assessed in a separate contrast against all correct outcomes. To test for any univariate responses related to the immediate goal, we performed an analogous analysis using the trial start regressors which were individuated based on the immediate goal. For example, the regressor ‘trialStartHouseNext’ was associated with the beginning of every trial where the farmhouse was the immediate goal location. To assess the univariate signal associated with the farmhouse immediate goal, we performed a contrast between this regressor and all other trial start regressors.

### Representational similarity analysis (RSA)

As before, a GLM was fit to the realigned functional images. The following events were modeled with impulse regressors: trial onset (information screen), key press to advance to the decision screen, the prompt and immediately subsequent action (modeled as a single regressor), the onset of the outcome screen, and the termination of the outcome screen. The RSA analysis used beta maps derived from the regressors marking trial onset, prompt/response, and outcome screen onset.

Each of these regressors (except those used in error trials) were further individuated by the (start state, next state, final goal) triple constituting the goal path. There were 8 distinct trial types starting in each state. Each state could serve as the starting point of two single-step sequences (in which the key and treasure chest are in the same location) and four two-step sequences (in which the key and treasure chest are in different locations). Each state could also be the midpoint of a two-step sequence with the treasure chest located in one of two adjacent states. With three regressors used for each trial, there were 4 starting states * 8 trial types * 3 time points = 96 total patterns used to create the Representational Dissimilarity Matrix (RDM) in each searchlight region, where cell x_ij_ in the RDM is defined as one minus the Pearson correlation between the ith and jth patterns. Values close to 2 therefore represent negative correlation (high representational distance) while values close to 0 indicate a positive correlation (low representational distance).

### Model RDMs

**Figure 6:**
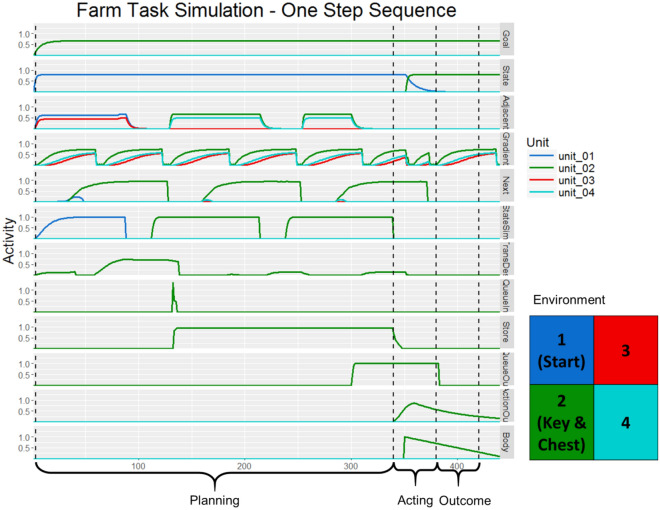
Model activity during a simulated one-step sequence of the Treasure Hunt task. The competitive queueing module first loads a plan and then executes it sequentially. State activity shows that the agent remains in state 1 for the first half of the simulation, while simulated-state (StateSim) shows the state transition the agent simulates as it forms its plan. Adjacent-states (Adjacent) receives input from stateSim which, along with goal-gradient (Gradient) activity determines the desired next state and therefore the appropriate transition to make. The plan is kept in queue-store (Store) which receives a burst of input from queue-input (QueueIn) and finally executes the plan by sending output to queue-output (QueueOut) which drives the motor system. The vertical dashed lines indicating the different phases of the simulation used in the creation of the model RDMs. For each layer, activity within each period was averaged across time to form a single vector representing the average pattern for that time period in the trial type being simulated. The bounds of each phase were determined qualitatively. The planning period is longer than the acting and outcome periods because the model takes longer to form a plan than execute it or observe the outcome.

**Figure 7:**
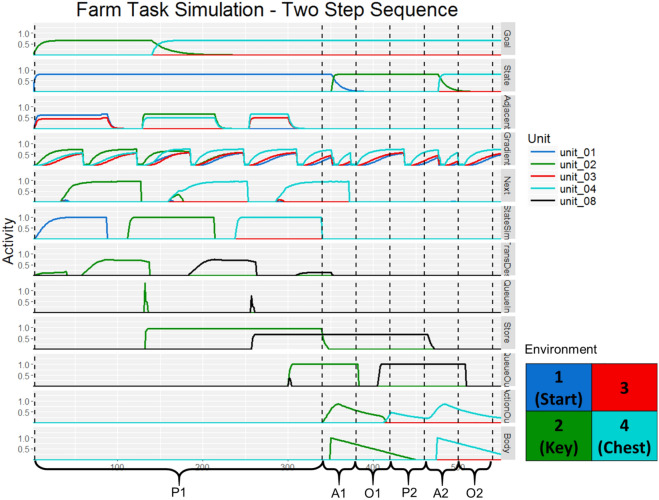
Model activity during a simulated two-step sequence of the Treasure Hunt task. The competitive queueing module first loads a plan and then executes it sequentially. State activity shows that the agent remains in state 1 for the first half of the simulation, while simulated-state shows the state transitions the agent simulates as it forms its plan. Adjacent-states receives input from simulated-state which, along with goal-gradient activity determines the desired next state and therefore the appropriate transitions to make. The plan is kept in queue-store which receives bursts of input from queue-input and finally executes the plan by sequentially sending output to queue-output which drives the motor system. To force the agent to go to the appropriate intermediate state, goal activity first reflects the key location and then the chest location. The vertical dashed lines indicate time periods used when creating the RDMs for the two-step sequence simulations. The first three time periods correspond to the first trial in the sequence while the latter three correspond to the second trial in the sequence. Again, the first planning period is much longer due to the nature of the model dynamics. During the second “planning” period (P2), the plan was already formed as must have been the case in the actual experiment since on the second trials in a two-step sequence, no information was presented at the start of the trial and had to be remembered from the previous trial.

To derive the model-based RDMs, the GOLSA model was run on an analogue of the goal pursuit task, using a four state state-space with four actions corresponding to movement in each cardinal direction. The model layer timecourses of activity are shown in Figs. 6 and 7 for one- and two-step trials, respectively. The base GOLSA model is not capable of maintaining a plan across an arbitrary delay, but instead acts immediately to make the necessary state transitions. The competitive queue^83^ module allows state transition sequences to be maintained and executed after a delay, and was therefore necessary to model the task in the most accurate manner possible. However, the goal-learning module was not necessary since goals were externally imposed. Because participants had to demonstrate high performance on the task before entering the scanner, little if any learning took place during the experiment. As a result, the model was trained extensively on the state space before performing any trials used in data collection. To further simulate likely patterns of activity in the absence of significant learning, the input from state to goal-gradient (used in the learning phase of an oscillatory cycle) was removed and the goal-gradient received steady input from the goal layer, interrupted only by the state-change inhibition signal. In other words, the goal-gradient layer continuously represented the actual goal gradient rather than shifting into learning mode half of the time.

In the task, participants first saw an information screen from which they could determine the immediate goal state and the appropriate next action. This plan was maintained over a delay before being implemented. At the beginning of each trial simulation, the queuing module was set to “load” while the model interactions determined the best method of getting from the current state to the goal state. This period is analogous to the period in which subjects look at the starting information screen and plan their next move. Then, the queuing module was set to “execute,” modeling the period in which participants are prompted to make their selection. Finally, the chosen action implements a state transition and the environment provides new state information to the state layer, modeling the outcome phase of the experiment.

Some pairs of trials in the task comprised a two-step sequence in which the final goal was initially two states away from the starting state. On the second trial of such sequences, participants were not provided any information on the information screen at the start of the trial, ensuring that they had encoded and maintained all goal-related information from the information screen presented at the start at the first trial in the sequence. These pairs of trials were modeled within a single GOLSA simulation. The model seeks the quickest path to the goal, identifying immediately available subgoals as needed. However, in the task, the location of the key necessitated a specific path to reach the final goal of the treasure chest. To provide these instructions to the model at the start of a two-step simulation, the goal representation from the subgoal (the key) was provided to the model first until the appropriate action was loaded and then the goal representation shifted to the final goal (the chest). Once the full two-step state transition sequence was loaded in the queue, the actions were read out sequentially, as shown in Fig. 7.

A separate RDM was generated for each model layer. Patterns were extracted from three time intervals per action (six total for the two-step sequence simulations). Due of the time required to load the queue, the first planning period was longer than all other intervals. For each simulation and time point, the patterns of activity across the units were averaged over time, yielding one vector. Each trial type was repeated 10 times and the patterns generated in the previous step were averaged across simulation repetitions. The activity of each layer was thus summarized with at most 96 patterns of activity which were converted into an RDM by taking one minus the Pearson correlation between each pattern. Patterns in which all units were 0 were ignored since the correlation is undefined for constant vectors.

We looked for neural regions corresponding to the layers that played a critical role in the model during the acting phase in the typical learning oscillation since in these simulations the learning phase of the oscillation was disabled. We created RDMs from the following layers: current-state, adjacent-states, goal, goal-gradient, next-desired-state, desired-transition, action-out, simulated-state, and queue-store. As a control, we also added a layer component which generated normally distributed noise (μ = 1, σ = 1).

### RSA searchlight

The searchlight analysis was conducted using Representational Similarity Analysis Toolbox, developed at the University of Cambridge (http://www.mrc-cbu.cam.ac.uk/methods-and-resources/toolboxes/license/). For each of these layer RDM, a searchlight of radius of 10 mm was moved through the entire brain. At each voxel, an RDM was created by from the patterns in the spherical region centered on that voxel.

An r value was obtained for each voxel by computing the Spearman correlation between the searchlight RDM and the model layer RDM, ignoring trial time periods in which all model units showed no activity. A full pass of the searchlight over the brain produced a whole-brain r map for each subject for each layer. Voxels in regions that perform a similar function to the model component will produce similar RDMs to the model component RDM and thus will be assigned relatively high values. The r maps were then Fisher-transformed into z maps ($z=\frac{1}{2}\mathrm{ln}\left(\frac{1+r}{1-r}\right)$). The z maps were normalized into the MNI template but were not smoothed, as the searchlight method already introduces substantial smoothing. Second level effects were assessed with a t test on the normalized z maps, with a cluster defining threshold of *p* < 0.001, cluster corrected to *p* < 0.05 overall. The cluster significance was determined by SPM5 and verified for clusters >  = 24 voxels in size with a version of 3DClustSim (compile date Jan. 11, 2017) that corrects for the alpha inflation found in pervious 3DClustSim versions^84^. The complete results are shown in Table 1.

## Supplementary Information

Supplementary Information.

## Data Availability

The GOLSA model code for the simulations is available at https://github.com/CogControl/GolsaOrigTreasureHunt. Imaging data are available from the corresponding author on reasonable request.
