# Supplementary material for: Foundations of human spatial problem solving
Source: Sci Rep. 2023 Jan 27;13:1485. doi: 10.1038/s41598-023-28834-3 (PMC9883268; doi:10.1038/s41598-023-28834-3)
Supplement: Supplementary file 1 — Supplementary Information. [file 41598_2023_28834_MOESM1_ESM.docx]

**RSA Comparison of Model and fMRI Data**

We compared the GOLSA model RDMs vs. the peak voxel RDM of the strongest match to each model layer, as specified in Table 1. The results are shown in Figure S1 below.

| **Model Layer** | **MNI coords** |
| --- | --- |
| Goal  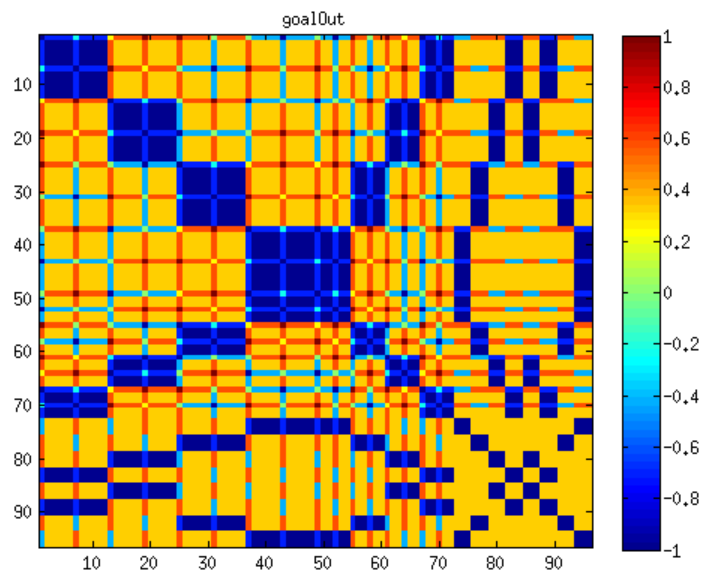 | -14, -86, 14 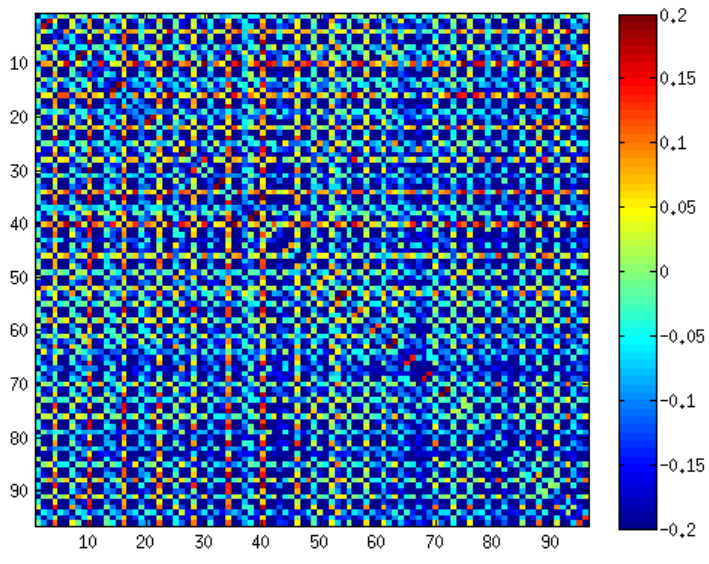 |
| Goal Gradient 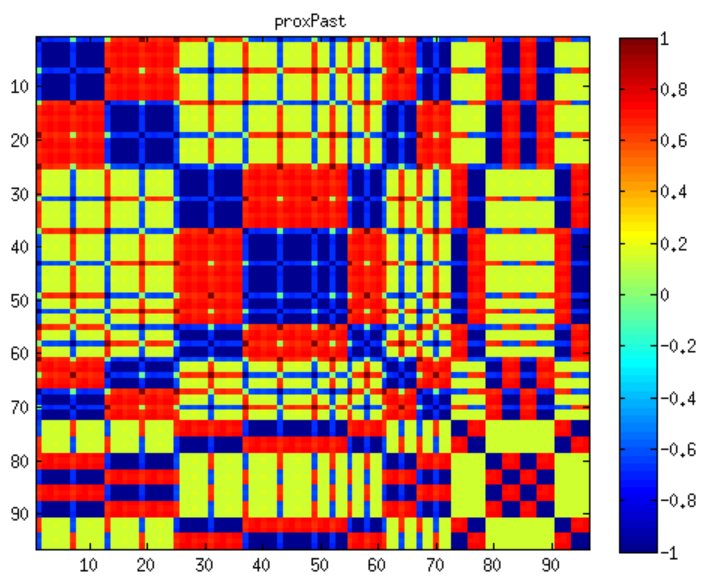 | 17, -79, -7  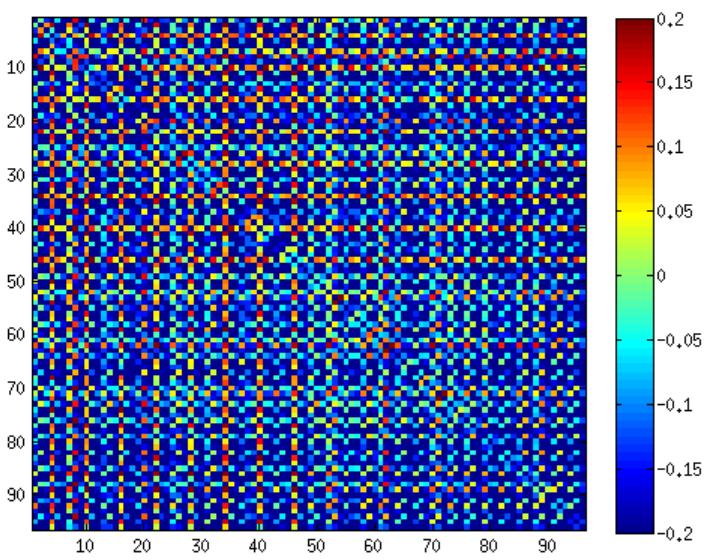 |
| Adjacent State 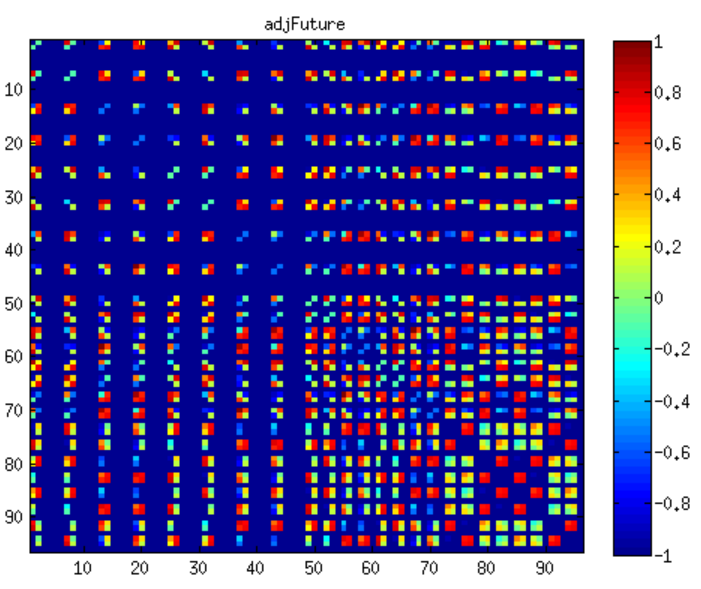 | 45, 24, -37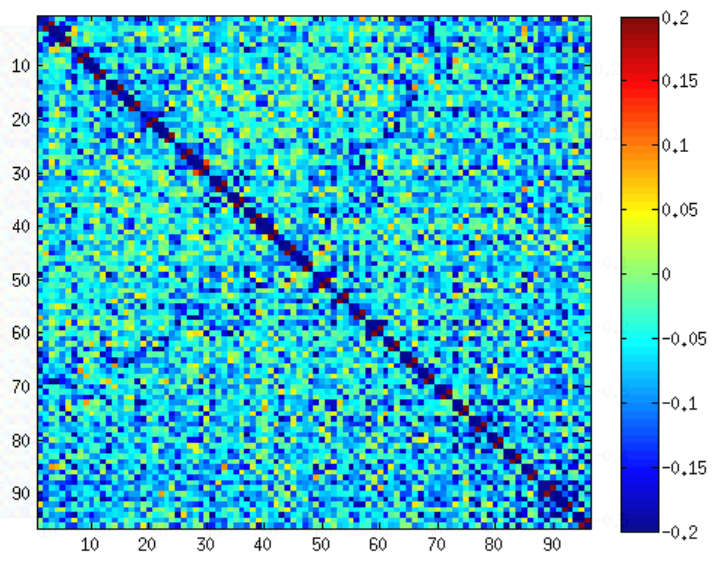 |
| Next Desired State 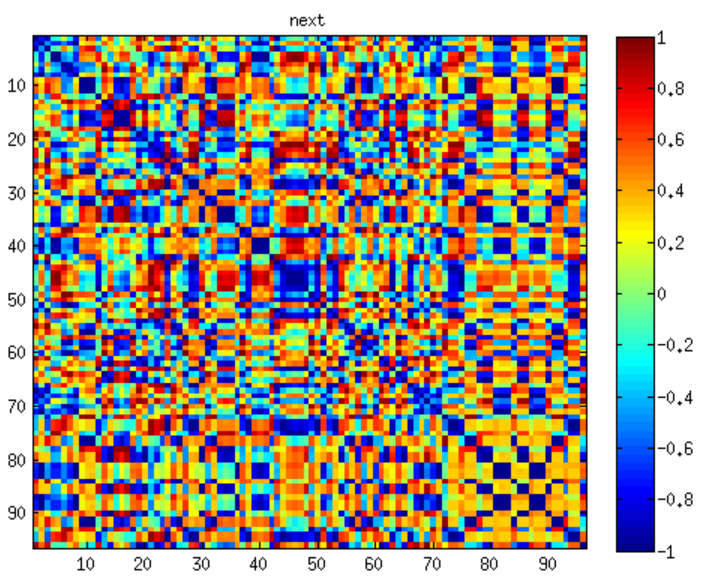 | 14, 31, -14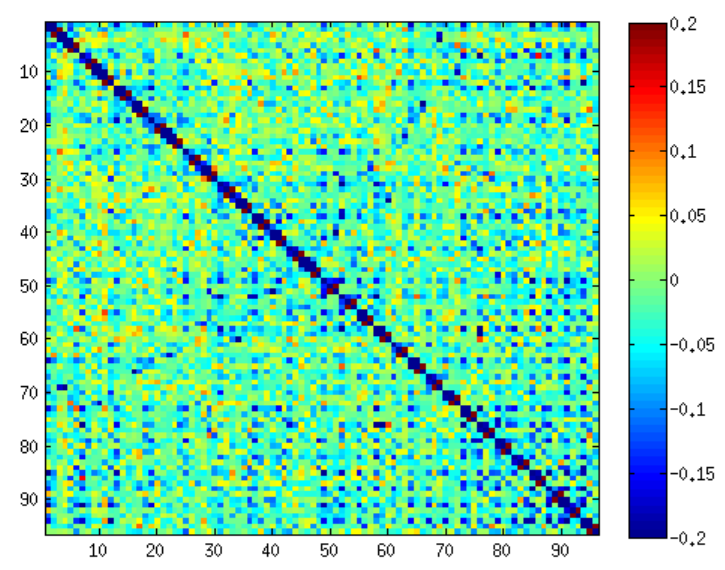 |
| Desired Transition 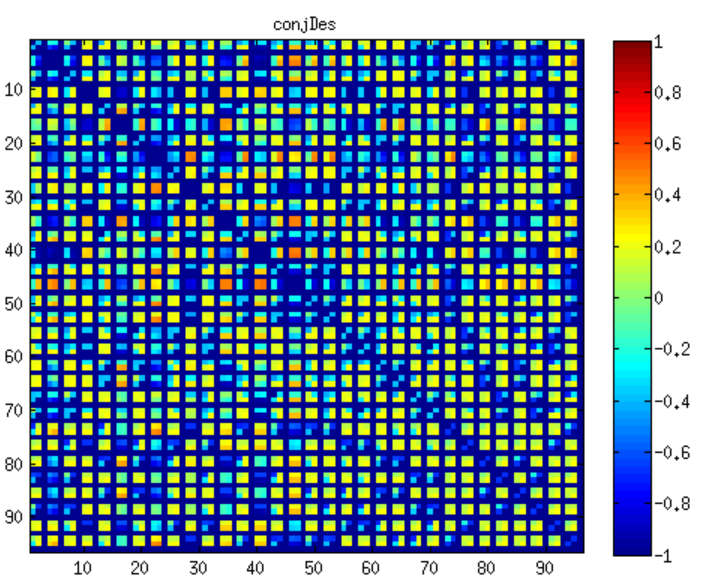 | -24, -7, 37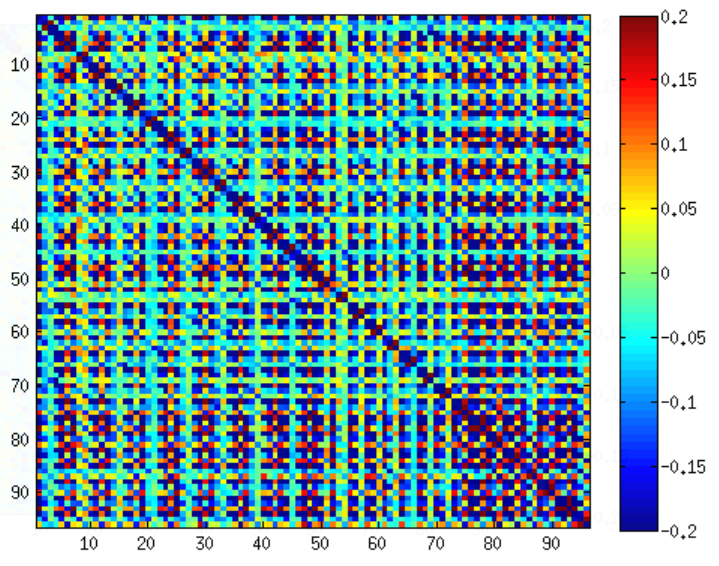 |
| Action Output 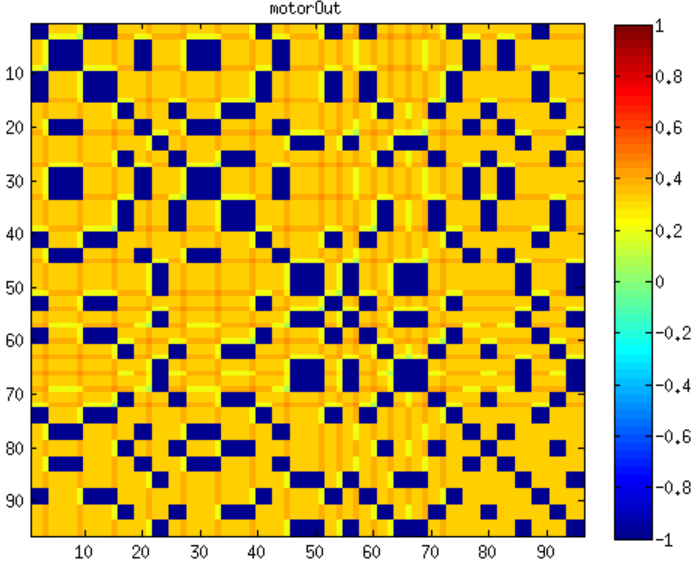 | -41, 0, 34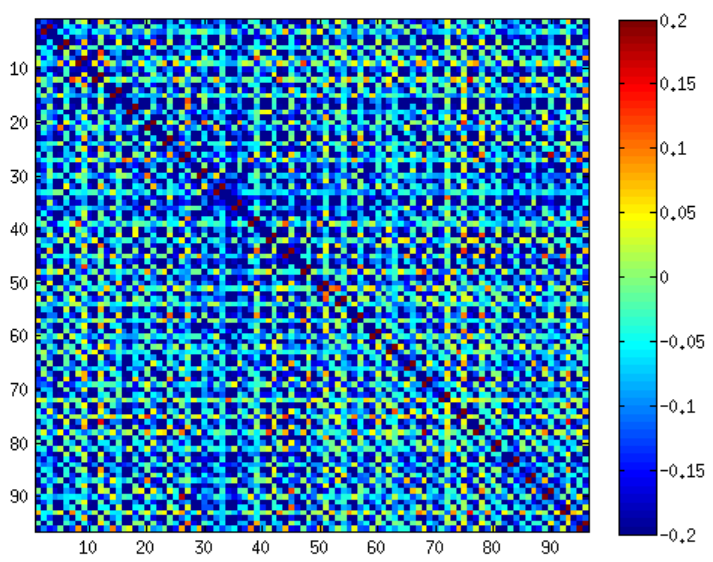 |
| Queue Store 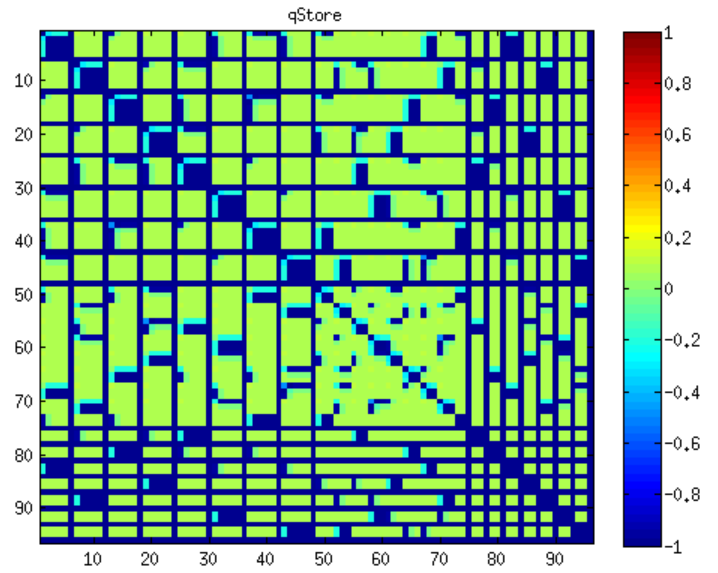 | 21, 58, 27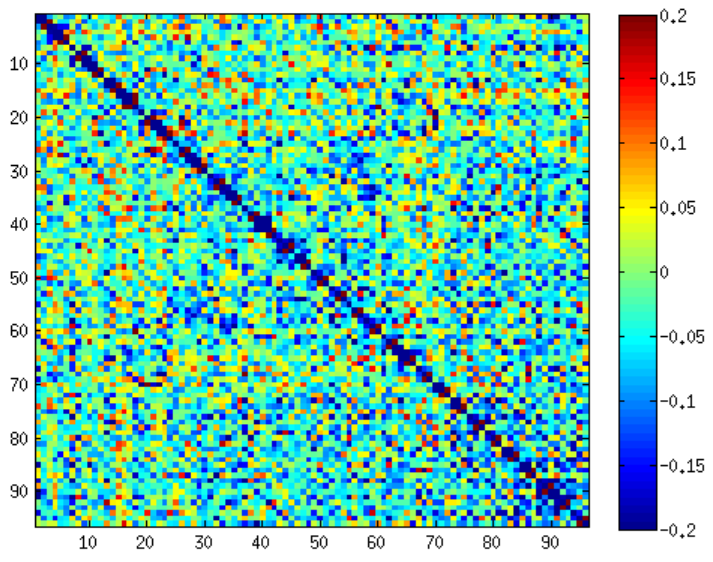 |
| Simulated State 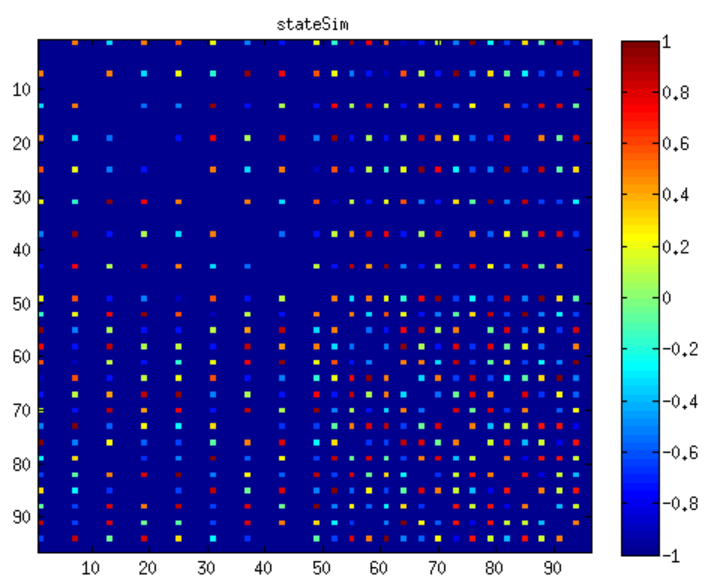 | 3, -83, -7  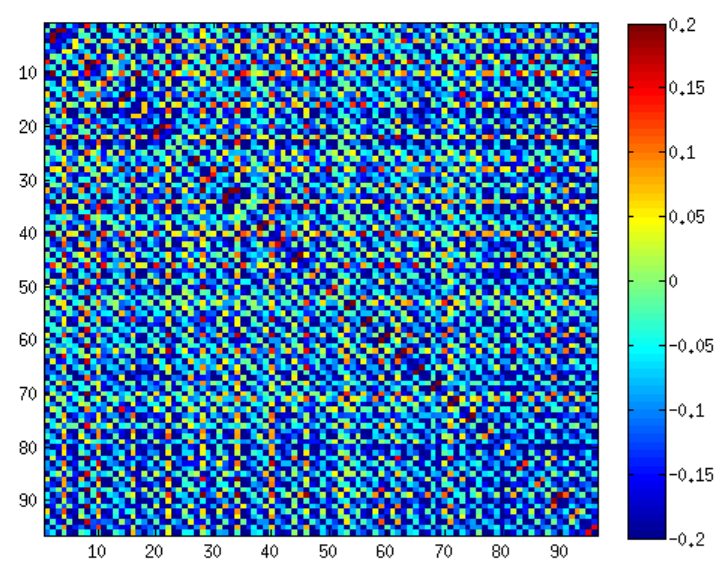 |
| State 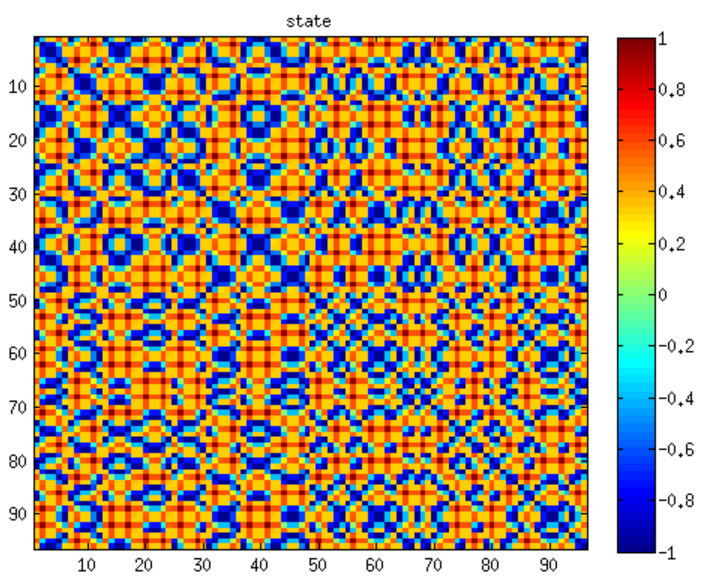 | -31, -14, 24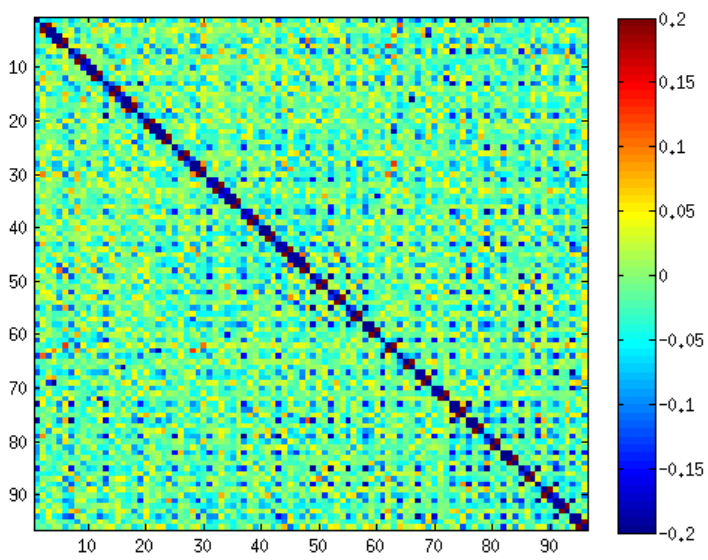 |

**Figure S1**. Comparison of GOLSA model RDMs (left) vs. fMRI peak-R RDMs (right). The fMRI RDMs were calculated from the MNI coordinate voxel that showed the strongest correlation with the corresponding model layer RDM as shown in Table 1.
